# Cannabis use during the early COVID-19 pandemic: Use patterns, predictors, and subjective experiences

**DOI:** 10.3389/fpsyt.2022.1037451

**Published:** 2023-01-10

**Authors:** Juliane Mielau, Simon Reiche, Daa Un Moon, Elisa Groß, Stefan Gutwinski, Felix Betzler, Amy Romanello, Dario Jalilzadeh Masah, Matteo Scicchitano, Roman Marek, Lasse Brandt, Ricarda Evens, Inge Maria Mick, Tomislav Majić, Christiane Montag

**Affiliations:** ^1^Department of Psychiatry and Psychotherapy, Charité Campus Mitte, Berlin Institute of Health, Charité – Universitätsmedizin Berlin, Corporate Member of Freie Universität Berlin and Humboldt-Universität zu Berlin, Berlin, Germany; ^2^Research Group Psychotropic Substances, Charité Campus Mitte, Charité – Universitätsmedizin Berlin, Corporate Member of Freie Universität Berlin and Humboldt-Universität zu Berlin, Psychiatric University Clinic at Hospital St. Hedwig, Berlin, Germany; ^3^Interdisciplinary Research Group “The Future of Medicine: Good Health for All,” Berlin-Brandenburg Academy of Sciences and Humanities, Berlin, Germany; ^4^German Sepsis Foundation, Berlin, Germany

**Keywords:** COVID-19, pandemic, SARS-CoV-2, cannabis, THC

## Abstract

**Background:**

Even in the early stages, global crises such as the COVID-19 pandemic lead to serious dislocations of social life, secondary adjustment reactions to external restrictions and individual concerns. Coping mechanisms may also include dysfunctional strategies like an increase of drug use. Considering the wide-spread use of cannabis, the aim of this study was to elucidate the interplay of social restrictions, psychopathology, concerns related to the pandemic in addition to the users’ experiences, motivations and consumption quantities during the early COVID-19 pandemic. It was presumed that cannabis intake would increase during the early phase of the crisis and that consumption quantities would be related to corona-related restrictions, concerns as well as subjective substance effects and psychopathology.

**Materials and methods:**

As part of an international, cross-sectional, internet-based survey (*N* = 5,049) available in five languages, consumption quantities and patterns of cannabis use in the early phase of the pandemic from April to August 2020 were examined. Participants retrospectively rated restrictions and concerns related to the pandemic, motives of cannabis use prior to and during 1 month the pandemic, and subjective consumption effects.

**Results:**

Cannabis use behavior showed no significant differences when consumption quantities prior and during 1 month after the COVID-19 outbreak were compared. Higher quantities of cannabis intake prior and during 1 month of the pandemic as well as more corona-related concern were associated with an increased perception of positive effects of cannabis during the pandemic. Predictors of its use during 1 month of pandemic were higher pre-pandemic consumption quantity, older age, quarantinization, a lesser degree of being affected by negative effects of the pandemic and a stronger subjective experience of corona-related positive effects of cannabis. Comparisons of the motives for cannabis intake in the pre-pandemic versus the pandemic period showed that all rationales for consumption were reported less frequently, except boredom.

**Conclusion:**

Frequencies of cannabis intake remained relatively stable in the early pandemic phase. Risk factors for increased use seem related to habitual consumption patterns that become more prominent under quarantinization. The use of cannabis as a dysfunctional coping strategy might not be amenable *via* self-report and should therefore receive special attention in clinical contexts.

## Introduction

The early phase of the spread of the coronavirus can be seen as exemplary for the beginning of a global crisis, being accompanied by considerable insecurity, restrictions of freedom and social dislocations. Healthy individuals as well as psychiatric patients are affected in terms of anxiety levels, symptoms of post-traumatic stress and depression (1), and access to treatment facilities might become limited. Individual reactions and coping attempts might also include dysfunctional strategies, such as the use of psychotropic substances. Scientific literature exploring possible changes in substance use disorders (SUDs) in times of pandemics is scarce (2). It is of special interest that the pandemic modifies the agenda of health professionals (3) and the treatment of SUDs appears to be immensely affected by COVID-19-related restrictions due to its mostly elective access to inpatient care, the voluntary basis of therapy and the transitional closure of outpatient facilities. Moreover, patients with SUDs are at higher risk of complications during treatment of SARS-CoV-2 infection, mainly because of pre-existing lung conditions, co-occurring comorbidities and, in many countries, limited access to public health facilities (4). For these reasons, there is an urgent requirement to highlight alterations caused by the pandemic. Patterns of usage of illicit and non-illicit drugs need to be clearly understood to effectively provide adapted drug treatment facilities for a vulnerable patient group. The global drug survey (GDS) as well as the European Monitoring Center for Drugs and Drug Addiction (EMCDDA) trendspotter briefing provided relevant details on the development on drug use until June 2020 (5, 6). The latter study reported that alcohol use became more frequent, being consumed in larger quantities, in non-social contexts and was combined with an increased use of prescribed medicines such as benzodiazepines. Conflicting international literature stating decreased alcohol consumption amounts during the beginning of the pandemic were published in 2020 and the following year (7–9). Boehnke et al. stated that more than 50% of participants recruited using Amazon Mechanical Turk *via* Cloud research either started using or increased use of medications or substances (most evident for alcohol and sleeping aids) because of the COVID-19 pandemic (10). Cannabis is a common substance consumed in social as well as non-social contexts. It has been proclaimed that on-going cannabis use results in poorer mental and physical health outcomes (11–15) which seems particularly relevant during ongoing restrictions of daily life.

Aim of this multi-lingual internet survey was to elucidate changes of cannabis consumption behaviors related to the early phase of the SARS-CoV-2 pandemic and associated stressors, to gain insight in motivations and reasons for use, to delineate changes in the subjective experience of cannabis use, and to assess possible interactions of its intake with parameters of mental health.

We hypothesized that cannabis intake would increase during the pandemic compared to the prior period. In addition, it was assumed that quantitative parameters and changes in intake would be related to negative sequelae and subjective concerns aroused by the COVID-19 crisis, and to corona-related positive effects of cannabis as well as psychopathology. Moreover, it was supposed that the consumption quantity during the early pandemic can be predicted by the pre-existing consumption rate, corona-related concerns, self-perceived negative effects of the pandemic, corona-related positive effects of cannabis intake and quarantinization. It was also hypothesized that participants being quarantined would report higher consumption rates and report different subjective effects of cannabis compared to persons who did not have to undergo this measure. On an exploratory basis, we aimed at determining changes of motivations for cannabis use before and during the pandemic. It was hypothesized that social or interactional contexts would be less relevant as common subjective motives for cannabis use during the pandemic, but that other reasons, such as regulation of affective states, would gain importance.

## Materials and methods

The study was approved by the Institutional Review Board (ethics committee) of the Charité – University of Medicine Berlin. A task force of psychiatrists and psychologists with experience in treatment of SUDs constructed an online-survey that was internationally available from April 30th until August 25th, 2020 in the following languages: German, English, Korean, Italian, Spanish. The survey was announced on social media, internet platforms such as Erowid, and mailing lists. The first section assessed the current situation in terms of the pandemic, related public conditions and their subjective experiences. Quantitative data regarding consumption patterns prior to the COVID-19 outbreak and during the last 4 weeks preceding the study participation were obtained and basic sociodemographic parameters were retrieved.

### Participants

Participants aged at least 18 years, being fluent in one of the aforementioned languages, needed approximately 25 min for anonymous participation. Usage of cannabis in 2019 and/or 2020 was defined as the main inclusion criterion. Demographic questions included age, gender, country of residence and current quarantine as well as an item on being temporarily quarantined during the last month. Current health situation (symptoms of coughing, fever or cold etc., COVID-19 test result in the past), social and professional situation were also asked. Subjects rated the quality and price of the illicit substance and were asked to rate supply difficulties during the pandemic compared to the prior period on a 5-point Likert scale (1-never; 2-rarely; 3-sometimes; 4-frequently, 5-very frequently).

### Measures

#### Symptom checklist-short version-9

All participants completed the 9-item short version of the Symptom-Check-List-90-R as extracted by Prinz et al. in 2008 (16) and constructed by Klaghofer and Brähler (17). The SCL-90-R is a 90-item self-report measure used to assess general psychopathology and overall psychological distress (18). The Symptom Checklist-short version-9 (SCL-K-9) included 3 items on depressive symptoms and one item for each of the following dimensions: anxiety, phobic anxiety, somatization, interpersonal sensitivity, paranoid ideation, hostility (19). Each of the nine items was rated on 5-point-Likert scales, whereby participants were asked to base their rating on the last 7 days (as compared with symptomatology prior to COVID-19 crisis).

#### Quantity of cannabis consumption

Moreover, participants rated their cannabis consumption frequencies on a 6-point-Likert scale (1–less than once per month; 2–circa once per month; 3–two to three times per month; 4–two to three times per week; 5–at least four times per week; 6–daily). A consumption change score was added to indicate the difference of consumption quantities pre-post-pandemic (i.e., negative values indicate an increasing consumption frequency and vice versa).

#### Motivations for cannabis consumption

To investigate the motives for cannabis use prior to as well as during the pandemic, respondents were asked to complete closed questions (yes/no) on the following possible rationales behind the respective substance usage: relaxation, partying, curiosity, self-experience, spiritual growth, numbing of anger/sadness, numbing of tension/inner emptiness, prescription by a physician, intensification of life, boredom, anesthetizing oneself, pleasure, cure of physical illnesses, cure of mental illnesses, encouragement by consuming friends/family members.

#### Negative effects of the pandemic

A sum score for negative effects of the pandemic comprised the following four items: isolation (“How strongly have you felt socially isolated during the last 4 weeks?”), awareness of media (“How intensely did you follow the news on COVID-19 during the last 4 weeks?”), restriction of life (“How severely did you restrict your way of life during the last 4 weeks?”), leaving the house (“How frequently have you left your house during the last 4 weeks?”). The items isolation and restriction of life were rated on the following 5-point Likert scale: 1- not at all; 2-very mild; 3-partially; 4-moderate; 5-severe. The item leaving the house was also rated on a 5-point Likert scale (1-much less frequently; 2-less frequently; 3-neither less or more frequently; 4-more frequently; 5-much more frequently). The item awareness of media has been handled in a similar manner (1-not at all; 2-rarely; 3-sometimes; 4-frequently, 5-very frequently). Recoding of the item leaving the house was executed prior to sum score calculation. Moreover, participants were asked whether they were currently quarantined.

Corona-related concern was assessed on a 4-point Likert scale (“How severely concerned have you felt during the last 4 weeks due to the COVID-19 developments?”) from “not at all” to “very severe.” Additionally, participants were invited to specify their concerns, whereby multiple responses were allowed. The following 14 concerns were available for selection: concern about COVID-19 infection of friends, family or oneself; death of oneself or many people in a short time span; overburdening of health systems; missing capacity for treatment of other diseases; oneself or others lacking social contacts; development of psychic symptoms; threat of an economic crisis; development of a political crisis; intensification of international conflicts; loss of income or jobs; difficulties in acquisition or financing of (illicit) substances; withdrawal; disadvantage with regard to inpatient treatment; other concerns to be specified by participants. A “corona concern” sum-score was created by adding all concern items and division by number of concern items thereafter. A low “corona concern” sum-score indicates less concerns due to impacts of COVID-19 crisis.

#### Cannabis effects

Ratings of seven items regarding differences in physical and mental effects of cannabis usage during the month of the pandemic as compared with previous experiences with the substance were obtained utilizing 5-point Likert scales (1-much less; 2-less; 3-equally; 4-more; 5-much more; 6-I do not know), respectively. Physical and mental effects considered in this survey were relaxation, intensification of taste, sexual pleasure, more profound knowledge of one’s life and the world, sense of threat, fear of somatic illnesses, fear for the future. The answer option “I do not know” was eventually not considered in the analysis.

#### Corona-related positive effects of cannabis intake

The items mastery (“Cannabis currently helps me to handle the COVID-19 crisis”), sense-giving (“Cannabis helps me to give a meaning to the COVID-19 crisis”) and handle isolation (“Due to cannabis consumption I can cope with social distancing during the last 4 weeks”) were added to create a sum score to account for experienced positive effects of cannabis intake participants, respectively rated the items mastery and sense-giving on 5-point Likert scales (1-totally disagree; 2-partially agree; 3-mostly agree; 4-agree; 5-strongly agree). The item handle isolation war also rated on a 5-point Likert scale (1-much worse; 2-worse; 3-neither worse or better; 4-better; 5-much better).

### Statistics

IBM PASW Statistics 26 was used to execute statistical calculations. Normal distribution was determined utilizing the Shapiro–Wilk test. Statistical significance was defined at a two-sided *p* < 0.05. Descriptive statistics comprise the calculation of frequencies, mean values and standard deviations of parameters relating to cannabis consumption. To compare cannabis usage prior to the pandemic versus up to 1 month preceding survey participation paired comparisons between variables were undertaken using Wilcoxon’s test. In order to investigate associations between quantitative cannabis intake and corona-related positive effects of cannabis, negative sequelae and concerns related to the COVID-19 crisis non-parametric Spearman correlational analyses were chosen. According to Cohen, effect sizes were classified as follows: weak: *r*(Spearman) >0.1, moderate: *r*(Spearman) >0.3, strong: *r*(Spearman) >0.5 (20). The impact of quarantinization on the experience of cannabis effects, corona-related positive effects of cannabis intake and consumption quantities was investigated using Mann–Whitney-U- and Wilcoxon tests. An ordinal logistic regression was performed using the cannabis consumption during 1 month of the pandemic as the dependent variable and gender and quarantinization as categorical independent variables. The following covariates entered the calculation: age, cannabis consumption quantities prior pandemic, corona concern sum score, sum score for negative effects of the pandemic and the sum-score of corona-related positive effects of cannabis. The predictors were tested *a priori* to exclude multicollinearity. McNemar tests were executed for all rationales behind consumption of cannabis for the pre-pandemic time span versus during the month of the pandemic.

## Results

### Demographics

The total sample of participants of the corona drug survey (*N* = 5,049) derived from 53 countries and contained 3,204 (63.5%) participants who consumed cannabis in 2019 or 2020. Table 1 contains the basic characteristics of the study participants. A total of 1,444 (45.1%) participants were under current quarantine at the time of the interview and 1,813 (56.7%) respondents had experienced quarantine during the preceding month. The compliance of participants regarding restrictive measures revealed the following distribution: none 55 (1.7%); not very compliant 125 (3.9%); partially compliant 804 (25.1%); rather compliant 1,533 (47.8%); very compliant 683 (21.3%). Symptoms of corona disease during the last 4 weeks were affirmed by 488 (15.2%) people, whereby only 133 respondents (4.2%) got a specific laboratory test. Six participants had a positive SARS-CoV-2 result; only one person during the preceding month. Supplementary Table 1 provides further information on pandemic changes of occupational conditions and associated fears of the subsample.

**TABLE 1:** Characteristics of respondents (*N* = 3,204).

	*N*	%
Age, years (mean SD)	24.08	(9.86)
**Gender**		
Male	1,827	57.5
Female	1,292	40.3
Diverse	50	1.6
Not specified	35	1.1
**Language used to complete survey**		
German	810	25.3
English	781	24.4
Spanish	1,354	42.3
Korean	0	0
Italian	285	8.1
**Education**		
Missing	129	4.0
Primary education	24	0.8
Secondary education	270	8.4
Technical college degree	220	6.9
A-level	921	28.7
Studies	1,294	40.4
Doctorate	52	1.6
Vocational training	293	9.1
**Occupational situation prior to pandemic**		
Seeking work	421	13.0
Unpaid household work	65	2.0
Paternity leave	22	0.7
Student job	590	18.6
Temporary employment	181	5.7
Fixed-term employment	353	11.1
Open-ended employment	881	27.7
Freelance	149	4.7
Self-employed	481	15.1
Retired	46	1.4
**Household members**		
None	442	13.8
One	542	16.9
2–3	1,261	39.4
>3	957	29.9
**Children**		
None	2,451	76.6
One	418	13.1
2–3	294	2.1
>3	35	1.1
**Current childcare situation**		
Children take care of themselves	113	15.3
Children at home, I mainly take care	160	21.7
Children at home, partner mainly takes care	42	5.7

### Concerns and positive effects of the pandemic

The concern regarding the corona pandemic in the sub-group of cannabis consumers was rated as follows: not at all 374 (11.7%); small 1,633 (52%); severe 905 (28.2%); very severe 292 (9.1%). 2,833 participants of the total sample specified their concerns. 65.7% (*N* = 1,861) of the respondents feared to acquire a corona infection or to have relatives being infected. 772 (*N* = 27.3%) participants were concerned to have a lethal course of illness. 41.7% (*N* = 1,182) of interviewees feared that the capacity for treatments of other illnesses will be limited and 46% (*N* = 1,304) anticipated associated mental issues. The concern for an economical exigency was raised by 2,097 people (74%), 1,113 participants (39.3%) expected a political crisis and 1,113 persons (39.3%) anticipated an intensification of international affairs. Loss of income or occupation was specified by 2019 (*N* = 71.3%) people. Financing or provision of substances was seen as a difficulty by 31.9% (*N* = 903), whereby 301 participants assumed to develop withdrawal symptoms. 68.5% of interviewees also reported a positive effect of the corona crisis: discharge of obligations (*N* = 1,024; 32%); new liberties (*N* = 516; 16.1%); new hobbies (*N* = 793; 24.8%); intensified contact to partner, family and/or friends (*N* = 880; 27.5%); more leisure time (*N* = 1,279; 39.9%); others (*N* = 257; 8%).

### Consumption associated conditions

Only 3 percent (*N* = 96) of the consumers stated to have used cannabis for the first time during corona crisis. *N* = 175 consumers had been seeking support due to their cannabis intake. A total of 14 participants consulted an emergency department, 42 reported inpatient treatment, 66 had psychotherapy, 37 chose withdrawal treatment, and 16 searched for other forms of support. The majority of the study participants stated that the quality (*N* = 1,900; 67.7%) and price (*N* = 1,412; 50.1%) of cannabis remained unchanged. Amongst those participants who affirmed a change in price and quality of cannabis, a minority noticed a diminished prices (*N* = 79; 2.8%) or improved quality (*N* = 220; 7.8%). Furthermore, limitations of cannabis supply rose when comparing pre-pandemic conditions with the time span of 1 month of the pandemic. Out of a total of 2,188 subjects, 44.1% (*N* = 966) specified that they never experienced supply difficulties prior to the pandemic. This statement was upheld by 628 individuals (29%) during pandemic. Likewise, the number of people stating very frequent supply limitations increased from 38 (1.7%) to 279 (12.9%) participants.

### Substance use behavior prior to COVID-19 outbreak versus during 1 month of pandemic

The majority of the participants (*N* = 1,229; 43.1%) denied to have altered the dosage of consumption whereby 890 (31.2%) lowered their dosage of cannabis within 1 month after corona outbreak. 25.6% (*N* = 730) of the sample increased their cannabis dosages. Faster redosing was reported by 601 patients (21.2%), whereas 420 participants (14.8%) declared a delayed urge for redosing. A total of 3,199 participants specified their consumption quantity prior the pandemic (mean = 4.15; SD = 1.92; median = 5) and 3,202 subjects quantified their consumption of cannabis during 1 month of the pandemic (mean = 4.14; SD = 1.89; median = 5). The Wilcoxon signed rank tests did not reveal a significant difference of the cannabis consumptions at the aforesaid time periods [*Z* = −0.838; *p* (2-tailed) = 0.402].

### Associations of reported corona-related positive effects of cannabis, negative sequelae and concerns related to the COVID-19 crisis with consumption quantities

As illustrated in Table 2, higher quantities of cannabis consumption prior and during 1 month of the pandemic were associated with an increased perception of positive effects of cannabis during the pandemic. The “corona concern” sum-score was positively correlated with the perception of negative effects of the pandemic (Spearman’s *r* = 0.269; *p* < 0.001; *N* = 2,834), and participants specifying more “corona concern” expressed more positive effects of cannabis during the pandemic (Spearman’s *r* = 0.103; *p* < 0.001; *N* = 2,178). However, no associations were found between “corona concern” and the quantitative cannabis consumptions or its changes during the pandemic.

**TABLE 2:** Spearman’s correlation coefficients of corona-related positive effects of cannabis, negative sequelae, and concerns related to the COVID-19 crisis with consumption quantities.

		Corona concern	Positive effects of cannabis during pandemic	Negative effects of pandemic
Consumption of cannabis prior to pandemic	*r*	0.011	0.357	−0.055
	*p*	0.570	**0.000**	**0.002**
	*N*	2,828	2,463	3,197
Consumption of cannabis during last month	*r*	0.015	0.484	−0.096
	*p*	0.424	**0.000**	**0.000**
	*N*	2,831	2,464	3,200
Consumption change score	*r*	0.002	−0.108	0.055
	*p*	0.897	**0.000**	**0.002**
	*N*	2,827	2,463	3,196
Sumscore- “corona concern”	r p N	1 – 2,836	0.103 **0.000** **2,178**	0.269 **0.000** **2,834**

Bold values indicates *P* < 0.05.

### Associations with psychopathology

Correlations between participants’ consumption quantities prior to the pandemic and general psychopathology as rated using the short version of the Symptom Checklist-short version-9 showed negligible effect sizes (cannabis consumption pre-COVID-19 pandemia: Spearman’s *r* = −0.051; *p* = 0.025; *N* = 1,903; cannabis consumption during 1 month of COVID-19 pandemia: Spearman’s *r* = −0.033; *p* = 0.145; *N* = 1,905).

### Predictors of the use of cannabis during 1 month of pandemic

Ordinal logistic regression analysis revealed that a significant improvement in fit of the final model over the null model was found [χ^2^(7) = 1,533.612; *p* < 0.001; Nagelkerke *R*^2^ = 0.538]. Whereas gender and corona-related concerns were not significantly contributing to the model, higher age, being placed in quarantine, a milder experience of the negative effects of the pandemic, a higher pre-pandemic cannabis consumption quantity and a stronger perception of corona-related positive effects of cannabis intake significantly predicted an increased intake during the pandemic. Odd’s ratios and 95%-confidence intervals are illustrated in Table 3.

**TABLE 3:** Predictors of the consumption quantity of cannabis during 1 month of the pandemic.

Variable	Coefficients	Standard error	Wald	*P*-value	OR	Confidence interval (95%)	
						Lower bound	Upper bound
Age	0.022	0.005	22.535	**<0**.**001**	1.022	0.013	0.013
Gender	-0.010	-0.010	0.13	0.910	0.990	-0.184	0.164
Negative effects of pandemic sumscore	-0.450	0.079	32.696	**<0**.**001**	0.638	-0.604	-0.296
Corona-related positive effects of cannabis	0.817	0.052	250.505	**<0**.**001**	2.264	0.052	0.918
No placement in quarantine	-0.264	0.092	8.198	**0**.**004**	0.768	-0.444	-0.083
Corona-concern sumscore	0.218	0.251	0.755	0.385	1.244	-0.274	0.710
Quantity of cannabis consumption prior pandemic	0.821	0.029	776.672	**<0**.**001**	2.272	0.763	0.878

Ordinal logistic regression analysis; OR, odd’s ratio. Bold values indicates *P* < 0.05.

### Impact of quarantine on consumption quantities and the experience of cannabis effects

Mann–Whitney-U-tests confirmed a significant difference between quarantined participants and users not being quarantined in their respective cannabis consumption quantities prior [*Z*(*N* = 3,206) = −5.965; *p* < 0.001] and during 1 month of the pandemic [*Z*(*N* = 3,208) = −3.27; *p* < 0.001]. Consequently, in both periods quarantined individuals were consuming cannabis more frequently. As portrayed in Table 4, it can be assumed that participants, who had been quarantined during this month of the pandemic, reported higher levels of relaxation, intensity of taste, knowledge of one’s life and the world and sexual pleasure in relation to cannabis use. Of note, the quarantined participants also affirmed sense of threat and fear of somatic illnesses to a larger degree.

**TABLE 4:** Impact of quarantine on the experience of cannabis effects, corona-related positive effects of cannabis intake and consumption quantities.

	Temporary quarantine	*N*	Mean rank	*Z*	*p* (2-tailed)
Relaxation	Yes	1,292	1154.60	−2.956	**0.003**
	No	953	1080.16		
	Total	2,245			
Intensification of taste	Yes	1,247	1087.91	−2.254	**0.024**
	No	889	1041.28		
	Total	2,136			
Sexual pleasure	Yes	1,017	965.38	−4.052	**0.000**
	No	835	879.14		
	Total	1,852			
Knowledge of one’s life and the world	Yes	1,217	1116.08	−6.347	**0.000**
	No	887	965.26		
	Total	2,104			
Sense of threat	Yes	1,007	833.39	−6.844	**0.000**
	No	794	986.74		
	Total	1,801			
Fear of somatic illnesses	Yes	1,061	907.26	−2.112	**0.035**
	No	793	954.58		
	Total	1,854			
Fear for the future	Yes	1,150	1004.83	−0.065	**0.948**
	No	860	1006.40		
	Total	2,010			
Positive effects of cannabis during pandemic	Yes	1,398	1344.61	−8.954	**0.000**
	No	1,068	1088.06		
	Total	2,466			

Mann–Whitney U test results for the experiences of cannabis effects, corona-related positive effects in quarantined and not quarantined users; bold values indicates *P* < 0.05.

### Rationales behind cannabis consumption prior to COVID-19 outbreak versus during 1 month of pandemic

Frequent motives for cannabis intake prior to corona restrictions were relaxation (*N* = 2,382; 82.6%), partying (*N* = 1,744; 60.5%), numbing of anger/sadness (*N* = 1,138; 39.5%), inner emptiness (*N* = 1,246; 43.2%) and pleasure (*N* = 2,206; 76.5%). During the month after corona outbreak all of the elected motives for cannabis consumption were chosen less frequently; especially motives such as partying (*N* = 639; 22.2%), curiosity (*N* = 217; 7.5%), pleasure (*N* = 1,697; 58.8%). Boredom was chosen as a reason for consumption of cannabis by 1,080 participants (37.4%) prior to and by 1,039 participants (36%) during 1 month of the pandemic. McNemar tests showed that there were significant differences regarding all rationales for cannabis consumption, except for boredom (see Table 5). *N* = 302 consumers who did not use cannabis as a means against boredom beforehand, reported cannabis consumption for this reason during the last 4 weeks. A total of 346 participants affirmed cannabis intake due to boredom prior to the pandemic but negated this rationale thereafter during the pandemic.

**TABLE 5:** Rationales for cannabis consumption prior versus during the pandemic.

Motives for consumption	*N* (prior pandemic)	*N* (during last month)	Two-tailed *P*-value*
Relaxation	2,382	1,909	<0.01
Pleasure	2,206	1,697	<0.01
Partying	1,744	639	<0.01
Numbing of tension/Emptiness	1,246	1.049	<0.01
Numbing of a anger/Sadness	1,138	920	<0.01
Boredom	1,080	1,039	0.117
Personal experience	1,050	724	<0.01
Curiosity	1,025	217	<0.01
Spiritual growth	902	673	<0.01
Due to consuming friends/Family	839	342	<0.01
Numbing of oneself	805	667	<0.01
Self-treatment of mental illness	647	511	<0.01
Intensification	612	414	<0.01
Self-treatment of physical illness	551	372	<0.01
Medical prescription	101	53	<0.01

*McNemar test.

## Discussion

This study examined aspects of cannabis consumption in non-clinical adults during the early phase of the COVID-19-pandemic. Main findings were that consumption quantities remained unchanged after the onset of the pandemic, and were not associated with corona-related concerns or psychopathology. Cannabis consumption amounts were, however, associated with subjectively positive effects of the substance, a milder experience of the negative effects of the pandemic, being placed in quarantine, more frequent pre-pandemic us, and higher age. Boredom was the only motive for its use that was maintained under pandemic conditions.

Although the hypothesis of an increased cannabis use during 1 month of the early SARS CoV-19 pandemic could not be confirmed, it was demonstrated that those individuals consuming greater quantities prior to COVID-19 outbreak were more likely to continue their consumption rate. Expectations of economical exigency were reported by nearly two thirds of the participants, and on the more individual level, of financing shortages of the substance were anticipated by circa one third of cannabis users. The number of users negating supply issues decreased by 15% during 1 month of the pandemic. However, the vast majority of the study participants stated unchanged quality and pricing of cannabis. Therefore, it seems rather unlikely that the absence of the foreseen rise of cannabis consumption during the early pandemic can be solely explained by alterations of its availability. The pandemic might have moreover encouraged particularly non-addicted cannabis consumers to adapt a more health-promoting life style. Our results are in line with another web-based survey completed by 3,632 unselected Belgian residents that yielded no significant changes in the consumption of cannabis when comparing pre-pandemic and pandemic quantities (21). Boehnke et al. found that more than a third of participants had increased, but another 25% had decreased cannabis use in the course of the pandemic (10). In contrast, 1,202 participants of an internet-based questionnaire of whom 76.7% claimed to use cannabis for mental health conditions, reported increased medicinal cannabis use by 91% since the COVID-19 pandemic (22). In the European study of the EMCDDA, 20% of participants reported a decrease and 40% an increase of usage of cannabis (6). Compared to users of other drugs, cannabis consumers were prone to replace their drug of choice by alcoholic beverages (6). It cannot be assumed that the respondents of our survey, originating from more than 50 countries, were exposed to identical pandemic-related confinement measures, and local accessibility of cannabis may have differed substantially. To summarize, our results regarding consumption quantities of cannabis in the early phase of COVID-19 pandemic are comparable to those of other large transnational study projects. The aforementioned, partly conflicting results either surveyed more defined sample groups (adolescent, residents of a certain country/region) and/or dealt with considerably smaller sample sizes.

In this study, more frequent cannabis use before and during the pandemic was associated with a stronger perception of positive effects of cannabis during the pandemic and marginally less awareness of negative effects of the pandemic. However, verbalization of greater corona-related concern was associated with more positively rated effects of cannabis during the pandemic, but not with quantitative features of use. Individuals who gathered experience in using cannabis as a means of regulating negative emotions or to facilitate coping with emotionally demanding situations, might have appreciated its relieving effect with increasing corona-related concerns. Interestingly, a sample of cannabis users in the US, who started their consumption during the pandemic, showed higher COVID-19-related worries than non-users and pre-pandemic users (23). Since our sample only contained 3% of first time cannabis consumers during the pandemic, one cannot draw conclusions concerning COVID-19-related worries in this subgroup. In partial accordance with our results, the authors described a reduction of social motives explaining variance in change of cannabis use, whereby, and similarly to the results of Benschop et al. from 2015, coping motives did not reveal a significant association with changes in cannabis use or symptom severity (24). Although thirty percent of the Dutch cannabis users of Cousijn’s survey experienced job losses and increased loneliness, contact with partners and families improved and their general mental health states remained unaltered (25). Since legal cannabis outlets were opened in Netherlands during lockdown, a great impact on the procurement of the substance cannot be assumed. On the other hand, addicted consumers might tend to downplay corona-associated as well as cannabis-related risks. The present study, however, yielded small respective correlation coefficients, and corona-related concerns were not associated with quantitative changes in cannabis use, indicating that cannabis use as a means for emotion regulation may be a neglectable phenomenon during the early pandemic.

Our survey results reveal that the consumption quantities were not associated with the self-reported psychological symptom load, which also argues against an increased use of THC for coping purposes. This seems counterintuitive, as a deterioration in depression and anxiety symptoms during lockdown was reported for circa one half of a Spanish SUD sample (26). Since our inclusion criteria and clinical self-report did not demand any clinical diagnoses, only a smaller share of participants might have exhibited full blown cannabis addiction or other mental disorders using cannabis for self-regulation or self-medication. Furthermore, this study did not explore non-medical forms of coping that could serve to attenuate symptoms and perceived distress. A reduced capability to perceive or identify symptoms or a bereft of critical thinking of those consumers fulfilling the addiction criteria must also be considered to elucidate our finding.

The present data allowed the identification of predictors of the cannabis intake quantity reported in the early pandemic. Quarantinization, older age, the stronger experience of corona-related positive effects of cannabis and a lower subjectively experienced severity of the restrictions of daily life were contributing to the prediction of higher cannabis consumption levels. Moreover, pre-pandemic usage quantities predicted cannabis consumption during 1 month of the pandemic, but the predictive value of quarantinization was preserved when this confounder was controlled for in the regression analysis. Results could be interpreted in the sense that older, more experienced or dependent users of cannabis consume the substance under pandemic conditions because of its known positive effects and even increase their consumption, when social contacts and activities are restricted, probably corresponding to their habitual problem-solving behavior. Younger, occasional users might not increase their use, when socially motivated consumption, e.g., at parties or festivals, is no longer an option. Of note, the appreciation of cannabis effects as helpful may rather drive consumption, while pandemic-related concerns did not seem to change use patterns and may also motivate health-conscious behavior. Quarantined individuals were more likely to report positive effects of cannabis (relaxation, intensification of taste, knowledge of one’s life and the world, sexual pleasure). In keeping with our result, Bartel et al. stated that Canadian participants in self-isolation used 20% more cannabis during the pandemic than those not being self-isolated (27). Self-isolation appeared to be an independent risk factor for higher cannabis use levels during the pandemic; irrespective of its usage in order to cope with depression during the pandemic (27). Interestingly, findings from a potentially overlapping subsample of our survey indicate a contrary effect of quarantinization on alcohol consumption that might be explained by the dominance of socially motivated use of this substance in non-clinical adults (28).

It can further be speculated that higher pre-pandemic cannabis use might even be associated with a higher risk of quarantinization, possibly due to shared risk factors such as impulsivity (29). Whereas during strict confinement in Spain decreases in cannabis use due to enhancement and social motives were evident (30), Salles et al. identified additional risk factors for cannabis use during the lockdown, such as age, concern for physical health, tobacco and alcohol consumption during lockdown, the pre-lockdown anger level and feelings of boredom during the restrictions (31).

In addition, our study explored changes in users’ motivations to consume cannabis before and during the weeks of the early pandemic. After the onset of the pandemic, social motives and intensification of pleasant feelings seemed to have become less salient than attenuation of negative emotions such as boredom. The most frequently reported rationales behind cannabis consumption during 1 month of pandemic were (1) relaxation, (2) pleasure, (3) numbing of tension/inner emptiness, (4) boredom, (5) numbing of anger/sadness, and (6) self-experience. McNemar testing proved that boredom as a motive of cannabis consumption was the only stable reason of consumption, and all other reasons were mentioned less frequently when comparing the pandemic and pre-pandemic statuses. Boredom has been discussed as a major motivation for cannabis use in psychotic patients as well as emerging adults (32, 33) and was a stable motive for cannabis use prior as well as during the pandemic in this sample. Results from studies investigating the use of other substances and respective consumption behaviors also highlight the role of boredom as a persistent motive (34–36). Vanderbruggen found the following most commonly stated motives for an increased cannabis consumption during COVID-19 lockdown measures: boredom, lack of social contacts, loss of daily structure, reward after a hard-working day, loneliness and conviviality (21). All other motives of cannabis use were chosen significantly less frequently during the pandemic. Motives relating to one’s expansion of consciousness, hedonism, creation of a collective experience were of lesser importance in the early pandemic. In accordance with previous findings concerning the relevance of cannabis intake for emotion regulation and coping (37, 38) the pre-pandemic motives of numbing of tension, anger and emptiness were reported frequently in our sample, but likewise significantly decreased during the early pandemic. It could be speculated that the contact restrictions and work place adjustments limit recreational use, but also the confrontation with interpersonal conflicts and therefore users are able to abstain from the euphoric or calming effects of cannabis. However, individual coping strategies (i.e., object resources, physical objects, personal resources, frustration tolerance) and their respective relevance in terms of substance use and distress relief were not specifically assessed in our study. Moreover, the degree of suffering during quarantine was not evaluated; thus it remains unclear whether individual coping mechanisms had to be implemented at all.

In summary, results of this survey disclose increases of cannabis use in association with corona-related restrictions and boredom and based on individual usage experiences, but do not substantiate motivations for use related to emotion regulation and coping with concerns, anxiety and psychopathology. However, a possible habitual reflection deficit might be more relevant in those cannabis consumers with high daily usage quantities, who might focus the positive effects of the substance, but tend to underestimate a possibly dysfunctional use of the substance for problem-solving.

## Limitations

In contrast to some previous studies focusing on illicit and legal substances in times of COVID-19 pandemic (5, 39, 40), this study revealed relatively stable cannabis consumption quantities, whereby cannabis users might idealize its occupying effect and in so doing, risk further social withdrawal. The present survey only assessed substance use during a 4-weeks period of the early pandemic. Therefore, long-term developments of substance usage in case of prolonged confinements and possible aggravation of access restrictions cannot be concluded. Results might, however, be exemplary for individual reaction modes and adaptation strategies that might similarly come into play in the early stages of other disaster situations. Biased sample selection occurred in the recruitment of anonymous cannabis users; hence full representation of the general population cannot be presumed. This sample contains fairly educated participants whereby users in difficult living conditions might be underrepresented in comparison to other recruitment strategies. Data collection was cross-sectional. Associations between pandemic-related measures, as well as reported effects of cannabis and changes of consumption quantities do not allow conclusions on causality. Moreover, pandemic-related restrictions and concerns, subjective effects of the illicit substance and rationales behind cannabis consumption were assessed using pre-set multiple-choice categories. These categories, in all likelihood, did not entail all subjectively important effects of cannabis and/or motives for usage. Despite these impediments, psychiatric carers should address a crucial need to assist cannabis users’ coping mechanisms to diminish substance-related harm. The roles of home hospitalizations, harm-reduction facilities, person-centered and integrated addiction care in people with SUDs will have to be redefined throughout and beyond the pandemic period (41).

## Data availability statement

The original contributions presented in this study are included in the article/Supplementary material, further inquiries can be directed to the corresponding author.

## Ethics statement

The studies involving human participants were reviewed and approved by the Institutional Review Board (Ethics Committee) of the Charité – University of Medicine Berlin. The patients/participants provided their written informed consent to participate in this study.

## Author contributions

JM: data analysis and interpretation, drafting and writing the article, critical revision of the article, and final approval of the version to be published. IM, SG, LB, and TM: conception and design of the work, interpretation, drafting the article, critical revision of the article, and final approval of the version to be published. FB: conception/design and critical revision of the article. RE: conception and design of the work and data analysis. SR, DM, DJM, MS, AR, and RM: conception and design of the work. CM: conception/design, data analysis, interpretation, drafting the article, critical revision of the article, and final approval of the version to be published. All authors contributed to the article and approved the submitted version.

## References

[1] Douglas PK Douglas DB Harrigan DC Douglas KM. Preparing for pandemic influenza and its aftermath: mental health issues considered. *Int J Emerg Ment Health.* (2009) 11:137–44. 20437844

[2] Guessouma SB Lachala L Radjacka R Carretiera E Minassiana S Benoita L Adolescent psychiatric disorders during the COVID-19 pandemic and lockdown. *Psychiatric Res.* (2020) 291:113264. 10.1016/j.psychres.2020.113264 32622172 PMC7323662

[3] Kontoangelos K Economou M Papageorgiou C. Mental health effects of COVID-19 pandemia: a review of clinical and psychological traits. *Psychiatry Investig.* (2020) 17:491–505. 10.30773/pi.2020.0161 32570296 PMC7324731

[4] Arya S Gupta R. COVID-19 outbreak: challenges for addiction services in India. *Asian J Psychiatr.* (2020) 51:102086. 10.1016/j.ajp.2020.102086 32315968 PMC7194862

[5] Winstock AR Davies EL Gilchrist G Zhuparris A Ferris JA Maier LJ *Global Drug Survey Special Edition on COVID-19: Interim Report.* (2020). Available online at: http://globaldrugsurvey.com (accessed August 15, 2022).

[6] European Monitoring Centre for Drugs and Drug Addiction [EMCDDA]. *Impact of COVID-19 on Patterns of Drug Use and Drug-related Harms in Europe, EMCDDA Trendspotter Briefing, Lisbon.* Lisbon: EMCDDA (2020).

[7] Garcia-Cerde R Valente JY Sohi I Falade R Sanchez ZM Monteiro MG. Alcohol use during the COVID-19 pandemic in Latin America and the Caribbean. *Rev Panam Salud Publica.* (2021) 45:e52. 10.26633/RPSP.2021.52 34025727 PMC8132959

[8] Kilian C Rehm J Allebeck P Braddick F Gual A Barták M Alcohol consumption during the COVID-19 pandemic in Europe: a large-scale cross-sectional study in 21 countries. *Addiction.* (2021) 116:3369–80. 10.1111/add.15530 34109685

[9] Callinan S Smit K Perez YM D’Aquino S Moore D Kuntsche E. Shifts in alcohol consumption during the COVID-19 pandemic: early indications from Australia. *Addiction.* (2020) 143:13826–8. 10.1111/add.15275 33006789 PMC7537267

[10] Boehnke KF McAfee J Ackerman JM Kruger DJ. Medication and substance use increases among people using cannabis medically during the COVID-19 pandemic. *Int J Drug Policy.* (2020) 92:103053. 10.1016/j.drugpo.2020.103053 33250438 PMC7685061

[11] González-Pinto A Alberich S Barbeito S Gutierrez M Vega P Ibáñez B Cannabis and first-episode psychosis: different long-term outcomes depending on continued or discontinued use. *Schizophr Bull.* (2011) 37:631–9. 10.1093/schbul/sbp126 19915168 PMC3080669

[12] Moore TH Zammit S Lingford-Hughes A Barnes TR Jones PB Burke M Cannabis use and risk of psychotic or affective mental health outcomes: a systematic review. *Lancet.* (2007) 370:319–28. 10.1016/S0140-6736(07)61162-3 17662880

[13] Grech A Van Os J Jones PB Lewis SW Murray RM. Cannabis use and outcome of recent onset psychosis. *Eur Psychiatry.* (2005) 20:349–53. 10.1016/j.eurpsy.2004.09.013 16018929

[14] Zammit S Moore TH Lingford-Hughes A Barnes TR Jones PB Burke M Effects of cannabis use on outcomes of psychotic disorders: systematic review. *Br J Psychiatry.* (2008) 193:357–63. 18978312 10.1192/bjp.bp.107.046375

[15] Linszen DH Dingemans PM Lenior ME. Cannabis abuse and the course of recent-onset schizophrenic disorders. *Arch Gen Psychiatry.* (1994) 51:273–9. 10.1001/archpsyc.1994.03950040017002 8161287

[16] Prinz U Nutzinger DO Schulz H Petermann F Braukhaus C Andreas S. The Symptom-Check-List-90-R (SCL-90-R) and the short versions of the SCL-90-R: psychometric analyses of inpatients with mental disorders. *Phys Med Rehab Kuror.* (2008) 18:337–43.

[17] Klaghofer R Brähler E. Konstruktion und teststatistische Prüfung einer Kurzform der SCL-90-R. *Z Klin Psychol Psychiatr Psychother.* (2001) 49:115–24.

[18] Derogatis L. *The SCL-90-R Manual. Clinical Psychometric Research Unit, Johns Hopkins University School of Medicine.* Baltimore, MD: Johns Hopkins University School of Medicine (1977).

[19] Prinz U Nutzinger DO Schulz H Petermann F Braukhaus C Andreas S. Comparative psychometric analyses of the SCL-90-R and its short versions in patients with affective disorders. *BMC Psychiatry.* (2013) 13:104. 10.1186/1471-244X-13-104 23537095 PMC3626675

[20] Cohen J. *Statistical Power Analysis for the Behavioral Sciences by Jacob Cohen (1988-08-12)–1. S. 79-81.* Milton Park: Routledg (1988).

[21] Vanderbruggen N Matthys F Van Laere S Zeeuws D Santermans L Van den Ameele S Self-reported alcohol, tobacco, and cannabis use during COVID-19 lockdown measures: results from a web-based survey. *Eur Addict Res.* (2020) 26:309–15. 10.1159/000510822 32961535 PMC7573904

[22] Vidot DC Islam JY Camacho-Rivera M Harrell MB Rao DR Chavez JV The COVID-19 cannabis health study: results from an epidemiologic assessment of adults who use cannabis for medicinal reasons in the United States. *J Addict Dis.* (2020) 39:26–36. 10.1080/10550887.2020.1811455 32933383 PMC9126187

[23] Rogers AH Shepherd JM Garey L Zvolensky MJ. Psychological factors associated with substance use initiation during the COVID-19 pandemic. *Psychiatry Res.* (2020) 293:113407. 10.1016/j.psychres.2020.113407 32827993 PMC7434361

[24] Benschop A Liebregts N van der Pol P Schaap R Buisman R van Laar M Reliability and validity of the Marijuana Motives Measure among young adult frequent cannabis users and associations with cannabis dependence. *Addict Behav.* (2015) 40:91–5. 10.1016/j.addbeh.2014.09.003 25240105

[25] Cousijn J Kuhns L Larsen H Kroon E. For better or for worse? A pre-post exploration of the impact of the COVID-19 lockdown on cannabis users. *Addiction.* (2021) 116:2104–15. 10.1111/add.15387 33394560 PMC8254730

[26] Blithikioti C Nuño L Paniello B Gual A Miquel L. Impact of COVID-19 lockdown on individuals under treatment for substance use disorders: risk factors for adverse mental health outcomes. *J Psychiatr Res.* (2021) 139:47–53. 10.1016/j.jpsychires.2021.05.006 34029833 PMC8769683

[27] Bartel SJ Sherry SB Stewart SH. Self-isolation: a significant contributor to cannabis use during the COVID-19 pandemic. *Subst Abus.* (2020) 12:1–4. 10.1080/08897077.2020.1823550 33044893

[28] Brandt L Evens R Reiche S Marek RM Moon DU Groß E Predictors of alcohol consumption among younger adults during the first phase of the COVID-19 pandemic. *Front Psychiatry.* (2021) 12:748158. 10.3389/fpsyt.2021.748158 34712158 PMC8546114

[29] Hicks TA Chartier KG Buckley TD Reese D The Spit for Science Working Group, Vassileva J Divergent changes: abstinence and higher-frequency substance use increase among racial/ethnic minority young adults during the COVID-19 global pandemic. *Am J Drug Alcohol Abuse.* (2022) 48:88–99. 10.1080/00952990.2021.1995401 35007453 PMC8855705

[30] Fernández-Artamendi S Ruiz MJ López-Núñez C. Analyzing the behavior of cannabis users during the COVID-19 confinement in Spain. *Int J Environ Res Public Health.* (2021) 18:11324. 10.3390/ijerph182111324 34769848 PMC8583130

[31] Salles J Yrondi A Marhar F Andant N Dorlhiac RA Quach B Changes in cannabis consumption during the global COVID-19 lockdown: the international COVISTRESS study. *Front Psychiatry.* (2021) 12:689634. 10.3389/fpsyt.2021.689634 34858218 PMC8632365

[32] Schaub M Fanghaenel K Stohler R. Reasons for cannabis use: patients with schizophrenia versus matched healthy controls. *Aust N Z J Psychiatry.* (2008) 42:1060–5. 10.1080/00048670802512016 19016094

[33] Goldstein AL Shifrin A Katz JL Iu LK Kofler D. Exploring the relationship between ADHD symptoms and daily cannabis consequences in emerging adulthood: the role of cannabis motives. *J Stud Alcohol Drugs.* (2021) 82:228–36. 33823970

[34] Evens R Reiche S Marek RM Moon DU Groß RE Romanello A Psychedelic experiences during the early COVID-19 pandemic: findings from an international online survey. *Front Psychiatry.* (2021) 12:732028. 10.3389/fpsyt.2021.732028 34803757 PMC8599818

[35] Bendau A Viohl L Petzold MB Helbig J Reiche S Marek R No party, no drugs? Use of stimulants, dissociative drugs, and GHB/GBL during the early COVID-19 pandemic. *Int J Drug Policy.* (2022) 102:103582. 10.1016/j.drugpo.2022.103582 35093679 PMC8730379

[36] Deng S Wang W Xie P Chao Y Zhu J. Perceived severity of COVID-19 and post-pandemic consumption willingness: the roles of boredom and sensation-seeking. *Front Psychol.* (2020) 11:567784. 10.3389/fpsyg.2020.567784 33041933 PMC7525206

[37] Bonara EE Goldstick JE Collins RL Cranford JA Cunningham RM Chermack ST Daily associations between cannabis motives and consumption in emerging adults. *Drug Alcohol Depend.* (2017) 178:136–42. 10.1016/j.drugalcdep.2017.05.006 28647681 PMC5548614

[38] Casajuana Kögel C López-Pelayo H Oliveras C Colom J Gual A Balcells-Oliveró MM. The relationship between motivations for cannabis consumption and problematic use. *Adicciones.* (2019) 33:31–42. 10.20882/adicciones.1221 31018002

[39] van Laar MW Oomen PE van Miltenburg CJA Vercoulen E Freeman TP Hall WD. Cannabis and COVID-19: reasons for concern. *Front Psychiatry.* (2020) 11:601653. 10.3389/fpsyt.2020.601653 33408655 PMC7779403

[40] Ashby NJS. Anonymized location data reveals trends in legal cannabis use in communities with increased mental health risks at the start of the COVID-19 pandemic. *J Addict Dis.* (2021) 17:1–8. 10.1080/10550887.2021.1886831 33595413

[41] López-Pelayo H Aubin HJ Drummond C Dom G Pascual F Rehm J The post-COVID era: challenges in the treatment of substance use disorder (SUD) after the pandemic. *BMC Med.* (2020) 18:241. 10.1186/s12916-020-01693-9 32731868 PMC7392642

