# Supplementary material for: Cannabis use during the early COVID-19 pandemic: Use patterns, predictors, and subjective experiences
Source: Front Psychiatry. 2023 Jan 10;13:1037451. doi: 10.3389/fpsyt.2022.1037451 (PMC9872100; doi:10.3389/fpsyt.2022.1037451)
Supplement: Supplementary file 1 [file Table_1.docx]

**Supplementary material:**

**Table 1b – Comparison of** **occupational conditions pre-pandemic and during one month of the pandemic**

|  | pre-pandemic | |  | During one month of pandemic | | |
| --- | --- | --- | --- | --- | --- | --- |
|  | N | % |  | N | % |  |
| Loss of occupation | - | - |  | 464 | 14.7 |  |
| Reduction of working hours | - | - |  | 677 | 21.5 |  |
| Fear of loss of employment | - | - |  | 407 | 12.9 |  |
| Notion of endangerment of one’s existence | - | - |  | 477 | 15.1 |  |
| Home office work | 570 | 18.1 |  | 1125 | 35.7 |  |
| Usage of usual work space | 1735 | 55 |  | 692 | 21.9 |  |
| Home schooling | 565 | 17.9 |  | 478 | 15.2 |  |
| Studying | 1061 | 33.7 |  | 783 | 24.8 |  |
| No employment | 681 | 21.6 |  | 1061 | 33.7 |  |
